# NapRNAdb: a multispecies repository and analytical platform for napRNA discovery and functional annotation

**DOI:** 10.1093/nar/gkaf1100

**Published:** 2025-11-03

**Authors:** Jiajia Xuan, Chunhua Xiao, Yonglei Luo, Shidong Tang, Junjie Pang, Zhirong Chen, Wanting Liu, Qing-Yu He

**Affiliations:** MOE Key Laboratory of Tumor Molecular Biology and State Key Laboratory of Bioactive Molecules and Druggability Assessment, Institute of Life and Health Engineering, College of Life Science and Technology, Jinan University, Guangzhou 510632, China; MOE Key Laboratory of Tumor Molecular Biology and State Key Laboratory of Bioactive Molecules and Druggability Assessment, Institute of Life and Health Engineering, College of Life Science and Technology, Jinan University, Guangzhou 510632, China; MOE Key Laboratory of Tumor Molecular Biology and State Key Laboratory of Bioactive Molecules and Druggability Assessment, Institute of Life and Health Engineering, College of Life Science and Technology, Jinan University, Guangzhou 510632, China; MOE Key Laboratory of Tumor Molecular Biology and State Key Laboratory of Bioactive Molecules and Druggability Assessment, Institute of Life and Health Engineering, College of Life Science and Technology, Jinan University, Guangzhou 510632, China; MOE Key Laboratory of Gene Function and Regulation, State Key Laboratory of Biocontrol, School of Life Sciences, Sun Yat-sen University, Guangzhou 510275, China; School of Pharmaceutical Sciences, State Key Laboratory of Traditional Chinese Medicine Syndrome, Guangzhou University of Chinese Medicine, Guangzhou 511400, China; MOE Key Laboratory of Tumor Molecular Biology and State Key Laboratory of Bioactive Molecules and Druggability Assessment, Institute of Life and Health Engineering, College of Life Science and Technology, Jinan University, Guangzhou 510632, China; MOE Key Laboratory of Tumor Molecular Biology and State Key Laboratory of Bioactive Molecules and Druggability Assessment, Institute of Life and Health Engineering, College of Life Science and Technology, Jinan University, Guangzhou 510632, China

## Abstract

Over 75% of the human genome transcribes noncapped RNAs (napRNAs), which typically function as noncoding RNAs in gene expression regulation. While short napRNAs (e.g. small RNAs) have well-defined transcriptomic roles, long napRNAs with diverse terminal modifications remain poorly characterized. To advance research, we developed NapRNAdb (https://bioinformaticsscience.cn/naprnadb), the first database dedicated to NAP-seq-derived napRNAs, enabling cross-species napRNA transcriptome profiling (40 species). This resource integrates 30 708 napRNAs derived from human and mouse NAP-seq with their expression profiles in multiple biological models, comprising 1016 ACA-napRNAs, 297 CD-napRNAs, 2302 Pol3-napRNAs, 157 polyA-pocket-ACA-napRNAs, 1536 stably expressed linear intron RNAs, 57 small nucleolar RNAs (snoRNAs)-intron napRNAs, 55 microRNA spacer-embedded RNAs, and 25 288 others. Leveraging evolutionary conservation analysis, NapRNAdb cost-effectively identifies 197 345 napRNAs spanning 8 categories in 38 species, with secondary structure and cross-species conservation for each molecule. Critically, NapRNAdb delivers the “PreTool” module for efficiently evaluating target sequence potential for napRNA processing via sequence similarity analysis. It also supports gene queries to verify participation in napRNA formation. Additionally, it systematically deciphers interaction networks linking napRNAs to RNA modifications, RNA-binding proteins, and disease-associated single nucleotide variants. Overall, NapRNAdb provides interactive interfaces to explore transcriptional, structural, expressional, and functional landscapes for napRNAs, facilitating comprehensive dissection of their biology while accelerating mechanistic insights into this field.

## Graphical Abstract

**Graphical Abstract:**
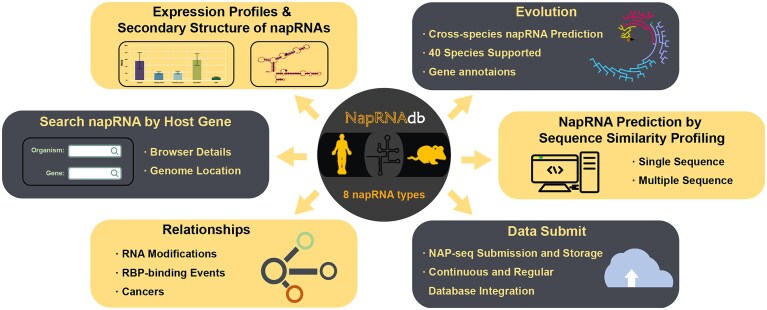

## Introduction

Over 80% of the human genome is transcribed by RNA polymerases into RNA, which is further processed into mature RNA comprising ∼5% capped RNAs (capRNAs) and ∼95% noncapped RNAs (napRNAs) [1, 2]. CapRNAs primarily consist of messenger RNAs (mRNAs) that are mainly translated into proteins and have been most extensively studied [2, 3]. In contrast, napRNAs typically function as noncoding RNAs (ncRNAs) in gene expression regulation [4–6], yet significant knowledge gaps persist regarding their genome-wide prevalence, mechanism, and regulatory function.

Advances in high-throughput sequencing technologies have promoted the identification and functional investigation of napRNAs. Conventional small RNA sequencing (sRNA-seq) and RNA sequencing (RNA-seq) are widely employed to study short napRNAs (<50 nt), leading to the discovery of functional small RNAs (sRNAs) such as microRNAs (miRNAs), p-element-induced wimpy testis (PIWI) -interacting RNAs (piRNAs), siRNAs, and 21U RNAs [7–9]. Extensive studies have established the roles of these short napRNAs in mRNA degradation, transcriptional silencing and translational repression [7–10]. In sharp contrast, current understanding of long napRNAs (≥100 nt) and those with diverse terminal modifications remains limited, largely due to the read length constraints of traditional RNA-seq [11]. Although pioneering studies reported a class of functional long napRNAs, such as long non-coding RNAs (lncRNAs) with snoRNA ends [12, 13] and hybrid mRNA–snoRNA [14], as broadly expressed in cells and tissues, technical limitations hindered comprehensive characterization, and functional studies of long napRNAs. This bottleneck was overcome with the recent development of NAP-seq, a full-length napRNA sequencing method compatible with Nanopore and Illumina platforms, enabling transcriptome-wide profiling of napRNA [15]. Applying NAP-seq, Liu *et al.* discovered structured and longer napRNAs with diverse terminal modifications in human and mouse, that typically missed by conventional sRNA-seq and RNA-seq [15]. According to the original study developing NAP-seq [15], stably expressed linear intron RNAs (sliRNAs) are defined as linear napRNAs overlapping entire intron regions and containing both 5′- and 3′-splice sites (5′-ss and 3′-ss). SnoRNA–intron napRNAs (snotrons) refer to napRNAs with one end matching the 5′/3′-end position of an intronic snoRNA and the other end aligning with the 3′/5′-end of an intron. MiRNA spacer-embedded RNAs (misRNAs) are linear napRNAs mapped to the sequences of miRNA spacer regions, with each end coinciding with one end of the two mature miRNAs. Pol3-napRNAs are transcribed by RNA polymerase III (pol III) and primarily processed from repetitive elements. They contain A/B box promoters and a 4U stretch at the 3′-end, and can fold into complex stem-loop structures. H/ACA napRNAs and C/D napRNAs refer to H/ACA box and C/D box snoRNAs identified from NAP-seq profiles, which reside within various known repetitive elements. Notably, these napRNAs are significantly longer than the canonical snoRNAs. PolyA-pocket-ACA napRNAs are H/ACA snoRNAs containing a poly(A) pocket. Although NAP-seq could discover diverse novel napRNAs, its reliance on custom RNA adapters and nanopore sequencing imposes high costs and technical barriers.

**Figure 1.**
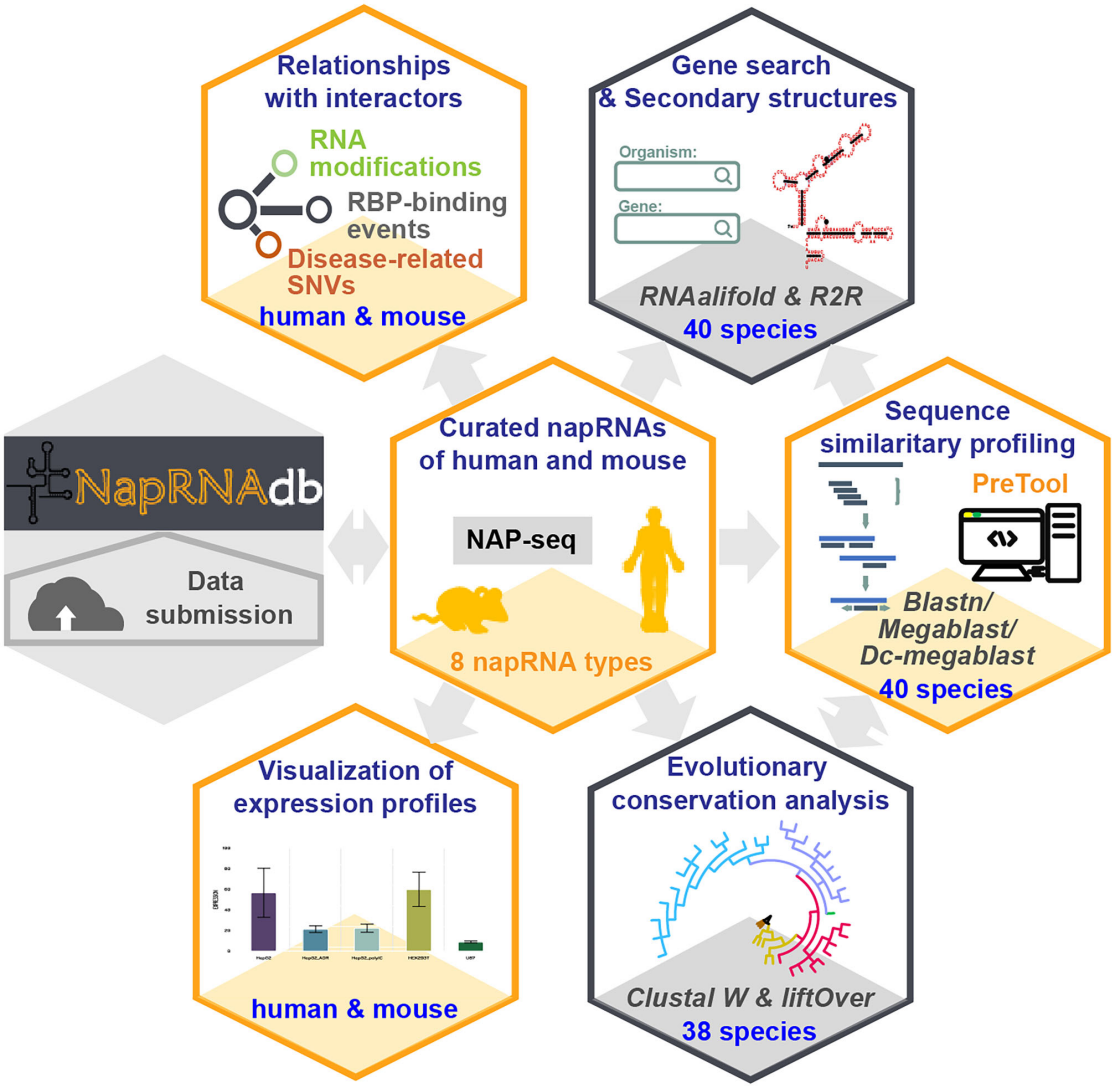
Graphic summarization for design and content of NapRNAdb database. NapRNAdb, as the first integrated repository dedicated to napRNA research, enables comprehensive investigation of napRNAs through multidimensional examination of their landscapes, structural architectures, expression profiles, and functional potentials. NapRNAdb also allows researchers to submit arbitrary napRNA datasets electronically to facilitate continuous updates and data management.

To address this, we created NapRNAdb (http://bioinformaticsscience.cn/naprnadb), the first dedicated database for integrating public napRNA resources and enabling cross-species or cross-cell lines prediction and functional exploration of potential napRNAs (Fig. 1). Firstly, we curated 30 708 napRNAs from fragmented studies in humans and mice, with expression profiles covering diverse cell types and physiological states. Leveraging the evolutionary conservation of repetitive elements generating napRNAs, we identified 197 345 candidate napRNAs across 38 species via sequence conservation analysis. Conveniently, users can rapidly determine whether a gene participates in formation of nascent napRNAs by gene name queries of 40 species. Critically, we developed a web-based tool named “PreTool”, which uses the curated known napRNAs as reference libraries to cost-effectively evaluate the transcriptional processing potential of any target sequence into napRNA using blastn, megablast and dc-megablast alignment modes. Additionally, systematic analysis of interactions between napRNAs and regulators, including RNA modifications, RNA-binding proteins (RBPs), and disease-associated single nucleotide variants (SNVs), explores their potential roles in gene regulation and disease. Finally, a “Submit” portal supports community submission of new napRNA datasets for continuous database updates. In summary, NapRNAdb serves as a dynamic platform to accelerate the deciphering of cross-species napRNA transcriptomes landscapes and their regulatory networks.

## Materials and methods

### Curation of napRNA sequences and expression profiles

We manually curated 16 992 human and 13 716 mouse napRNAs from the recent published literature [15]. These molecules were systematically classified into eight categories: H/ACA napRNAs (ACA-napRNAs), C/D napRNAs (CD-napRNAs), Pol III-transcribed napRNAs (Pol3-napRNAs), H/ACA snoRNAs harboring poly(A) pockets (polyA-pocket-ACA-napRNAs), sliRNAs, snotrons, misRNAs, and other novel napRNAs. leveraging experimentally validated data, we integrated expression profiles of these napRNAs across established cell models, including human HepG2, HEK293T, and U87 lines, as well as murine C2C12 cells.

### Genome sequences and annotations of 40 species

Genomic sequences and corresponding gene annotations for 40 species were retrieved from the UCSC Genome Browser [16]. To ensure consistency and accuracy in downstream analyses, annotations for each species were converted to a standardized format. Based on classification criteria defined by GENCODE [17] and Ensembl [18], genomic elements were categorized into six distinct biotypes: mRNA, lncRNA, sncRNA, pseudogene, repeat elements, and intergenic regions. Furthermore, gene features were classified into five types required for the analysis: 5′-untranslated region (5′-UTR), 3′-UTR, coding sequence, exon, and intron.

### Sequence similarity analysis

Sequence-dependent napRNA identification was implemented using BLAST (v2.16.0+) [19]. Curated human and murine napRNA sequences were first compiled to construct a reference alignment database using makeblastdb (parameters: -dbtype nucl -parse_seqids). Three computational models were then deployed for sequence alignment: Megablast for highly similar sequences (closely related species), discontinuous Megablast (dc-megablast), and blastn for divergent sequences (distantly related species). Users can customize parameters including the seed length, Max target sequences (Max_target_seqs), Expect value (E-value) threshold, Match/Mismatch scores, and Gap costs to optimize computational efficiency while maintaining alignment accuracy. A scoring system based on three key BLAST metrics, including percent identity (pident), expect value (e-value), and query coverage (qcovs), is used to evaluate and assign confidence levels to result entries. The specific criteria are as follows: (i) Score each metric according to Supplementary Table S1; (ii) Calculate the total score and classify the confidence level as High (total score ≥ 11), Medium (8 ≤ total score < 11), or Low (total score < 8).

The output fields include: qaccver (Query accession.version), saccver (Subject accession.version), Score, Confidence, pident (Percentage of identical matches), Length (Alignment length), Mismatch (Number of mismatches), Gap openings, qstart (Start position in query), qend (End position in query), sstart (Start position in subject), send (End position in subject), evalue (Expect value), bitscore (Bit score), qcovs (Query coverage per subject), sstrand (Subject strand), qseq (Aligned part of query sequence), sseq (Aligned part of subject sequence).

### Computational prediction of napRNA secondary structure

RNAalifold program in the ViennaRNA package (v2.4.18) [20] was used to predict the secondary structures of napRNAs. Coevolutionary visualization of napRNA structures was performed by R2R (v1.0.6) [21].

### Cross-species conservation analysis

NapRNA sequences of different species were aligned by ClustalW (v2.1) [22]. To improve the reliability of subsequent phylogenetic analysis, the resulting multiple sequence alignments (MSA) were stringently trimmed using TrimAl (v1.5.rev0) [23] with the parameters: -gt 0.8 -st 0.001 -cons 80. Based on the trimmed alignments, Maximum-Likelihood trees were constructed with IQ-TREE (v3.0.1) [24], while Neighbor-Joining trees were constructed using the R package ape (v5.8.1) [25]; homologous groups containing >4 sequences were assessed with 1000 ultrafast bootstrap replicates for branch support in both cases.

UCSC chain files and liftOver tool [16] from UCSC Genome Browser were employed for genomic coordinate mapping of homologous sequences among different species. Based on these resources, we applied standardized pipelines to annotate napRNAs in these species.

BLAST tool was employed for sequence homology analysis of napRNAs among different species. NapRNA sequences from mammalian and nonmammalian species were aligned to the pre-constructed reference library using dc-megablast (seed = 12) and blastn (seed = 7), respectively. To evaluate the confidence of the alignments, a scoring system was established based on the above three key BLAST metrics. Each metric was assigned a score from 1 to 4 (low to high) according to predefined thresholds (Supplementary Tables S2 and S3). The total score was then calculated and classified into three confidence levels: High (≥11), Medium [8–10], or Low (<8). Results failing to align with reference napRNAs were uniformly assigned a score of 2 (low confidence). To increase the credibility of annotated entries, cross-species candidate predictions were annotated with evidence tags: “C” for results based solely on evolutionary conservation analysis, “H” for those based on sequence homology, and “C,H” for entries supported by both.

### Association analysis of napRNAs with interacting factors

RNA modifications were downloaded from RMBase v3.0 [26], RBP-binding events that were supported by CLIP-seq derived from starBase [27], and SNVs were curated from COSMIC [28]. These datasets were converted to BED6 format and intersected with napRNA coordinates using BEDTools (v2.30.0) [29].

Regulatory pairs were classified into five categories based on the binding positions of interaction factors relative to napRNAs: [1] binding sites located ≤20 nt downstream of the 3′-end (3′-ss); [2] sites proximal to the 3′-end (≤20 nt) but classified as internal (3′-ss-internal); [3] sites located ≤20 nt upstream of the 5′-end (5′-ss); [4] sites proximal to the 5′-end (≤20 nt) but classified as internal (5′-ss-internal); and [5] binding sites >20 nt from both 5′- and 3′-ends (internal).

### Genome browser configuration

Genome browser was implemented using JBrowse (v1.16.11) [30]. Reference genome sequences and baseline gene annotations for human (hg38) and mouse (mm10) were obtained from GENCODE. NapRNA annotations were loaded as browser tracks in GFF3 format. Additionally, the following functional tracks were configured: RBP-binding sites and RNA modifications for human and mouse, as well as SNV sites for human (all in BED format). NapRNAs from the other 38 species were integrated as tracks after being mapped to the human or mouse genome via homologous mapping.

### Implementation of NapRNAdb

All processed data and analytical pipelines were archived in NapRNAdb, a comprehensive and flexible platform implemented using HTML5, PHP7, CSS3, and JavaScript. We employed multiple external packages and software to render and visualize database content, including the Bootstrap (v4.4.1) framework for responsive web interface design, MySQL (v8.0) for backend data storage and query operations, DataTables for dynamic tabular data presentation, Highcharts (v9.3.2) for interactive visualization of complex datasets, and Common Gateway Interface (CGI), R (v4.1), and Perl (v5.32) to support analytical workflows in the “PreTool” module.

## Database content and web interface

### Comprehensive browsing of various types of napRNA

**Figure 2.**
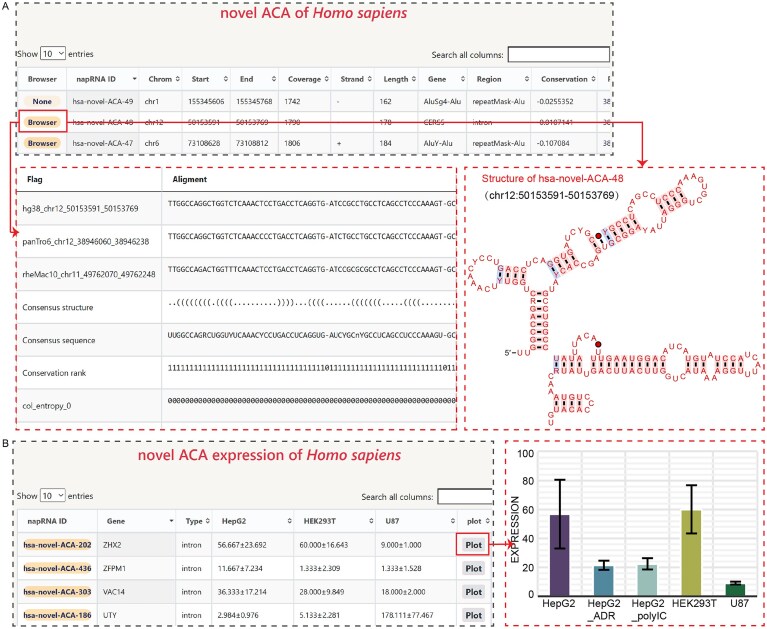
Details of the queried napRNA. (**A**) Secondary structure prediction of the queried napRNA (has-novel-ACA-48). (**B**) Expression profile of the napRNA in diverse human cell lines (HepG2, HEK293T, and U87).

We manually curated 30 708 napRNA molecules from recent publications, including 16 992 human and 13 716 mouse napRNAs. Based on host type, they were classified into: 1016 ACA-napRNAs, 297 CD-napRNAs, 2302 Pol3-napRNAs, 157 polyA-pocket-ACA-napRNAs, 1536 sliRNAs, 57 snotrons, 55 misRNAs, and 25 228 unclassified subtypes (Table 1). To enhance biological relevance, we annotated each napRNA with genomic coordinates, strand orientation, gene symbols, and genomic features using standardized annotation files. We further performed cross-species conservation of napRNAs and predicted their secondary structures. NapRNAdb enables users to inspect conserved sequences and structural maps in detail pages (Fig. 2A).

**Table 1.** Statistics of various napRNAs in human and mouse

napRNA	Human	Mouse
ACA-napRNAs	889	127
CD-napRNAs	226	71
Pol3-napRNAs	87	2215
polyA-pocket-ACA-napRNAs	157	0
sliRNAs	620	916
snotrons	35	22
misRNAs	26	29
novel-napRNAs	14 952	10 336

Furthermore, we developed an “Expression” module to integrate experimentally validated expression profiles of napRNAs from the recent published literature [15] across diverse cell lines and stress conditions—including human HEK293T, U87, and HepG2 cell lines; Adriamycin (ADR)- and Poly(I:C)-stimulated HepG2 cells; and murine C2C12 cells at four differentiation stages. All expression profiles feature interactive visualization with export options for multiformat figures (Fig. 2B).

### Cross-species recognition of napRNAs via evolutionary conservation profiling

Given that most napRNAs originate from evolutionarily conserved repetitive sequences, we performed sequence conservation and homology analyses across 38 species spanning 7 major phylogenetic clades: Aves, Euarchontoglires, Fish, Laurasiatheria, Mammals, Primates, and Sarcopterygii, following annotation methods previously established for Pol III-transcribed ncRNAs [31]. We identified 197 345 candidate napRNAs classified into eight phylogenetically conserved categories (Table 2). To enhance the reliability of the annotation entries, each candidate was explicitly labeled with conservation status relative to human or mouse orthologs and assigned evidence tags—“C” indicating support from sequence conservation, and “H” indicating sequence homology. To reduce false positives, a scoring system was developed based on sequence homology metrics between each candidate napRNA and reference napRNAs, including percent identity (pident), expect value (evalue), and query coverage (qcovs). Each candidate was assigned a score using this system and classified into one of three confidence levels according to the total score: High (≥11), Medium [8–10], or Low (<8). To facilitate access, these napRNAs were cataloged in the “Evolution” module with detailed genomic annotations, phylogenetic trees and reliable secondary structure maps. Additionally, NapRNAdb provides a “Gene” module enabling users to retrieve associated napRNA information by querying host genes in species of interest.

**Table 2.** Statistics of various napRNAs in 38 additional species

Species	ACA-napRNAs	CD-napRNAs	Pol3-napRNAs	polyA-pocket-ACA-napRNAs	sliRNAs	snotrons	misRNAs	novel-napRNAs
*Anolis carolinensis*	7	2	0	0	46	8	1	150
*Bos taurus*	98	69	2	1	407	24	17	2586
*Callithrix jacchus*	451	159	42	84	723	35	27	9536
*Canis lupus familiaris*	113	65	8	1	422	26	22	3117
*Cavia porcellus*	56	42	5	1	260	20	15	1598
*Chlorocebus sabaeus*	588	197	58	122	569	32	24	11 923
*Cricetulus griseus*	63	39	54	2	486	23	18	1854
*Danio rerio*	2	1	0	0	5	0	0	31
*Dipodomys ordii*	4	5	3	0	95	1	3	260
*Equus caballus*	145	107	12	1	618	29	22	4381
*Felis catus*	115	67	6	1	498	29	20	3283
*Gallus gallus*	10	9	0	0	88	9	4	226
*Gorilla gorilla*	724	234	86	155	885	43	31	14 937
*Heterocephalus glaber*	66	50	7	2	346	21	16	2146
*Macaca mulatta*	614	209	68	122	842	39	29	12 554
*Macropus eugenii*	13	7	1	1	80	6	3	335
*Microcebus murinus*	144	90	9	10	481	30	19	4167
*Monodelphis domestica*	26	13	4	1	163	12	5	664
*Nomascus leucogenys*	637	213	66	126	835	38	30	12 985
*Ochotona princeps*	25	16	2	1	133	16	9	1090
*Ornithorhynchus anatinus*	11	6	2	0	93	6	5	360
*Oryctolagus cuniculus*	47	31	2	1	251	19	13	1869
*Otolemur garnettii*	133	77	7	12	437	24	19	3099
*Ovis aries*	90	60	4	1	368	23	15	2558
*Pan troglodytes*	731	236	88	152	894	43	31	15 063
*Papio hamadryas*	622	215	67	124	833	41	28	12 717
*Pongo pygmaeus abelii*	702	230	81	153	885	41	30	14 476
*Rattus norvegicus*	62	46	223	1	597	20	27	2529
*Saimiri boliviensis*	419	159	34	73	707	33	25	8824
*Sorex araneus*	26	22	0	1	118	12	11	980
*Spermophilus tridecemlineatus*	79	55	7	1	403	23	14	2437
*Sus scrofa*	89	68	5	1	386	27	20	2714
*Takifugu rubripes*	3	1	1	0	18	2	1	48
*Tarsius syrichta*	135	92	11	9	512	27	20	3895
*Trichechus manatus latirostris*	114	84	5	1	504	27	20	3058
*Tupaia belangeri*	55	48	2	1	231	18	12	1724
*Vicugna pacos*	96	75	8	0	481	26	14	3259
*Xenopus tropicalis*	5	5	0	0	37	4	3	129

### A web-based system for napRNA prediction via sequence similarity profiling

**Figure 3.**
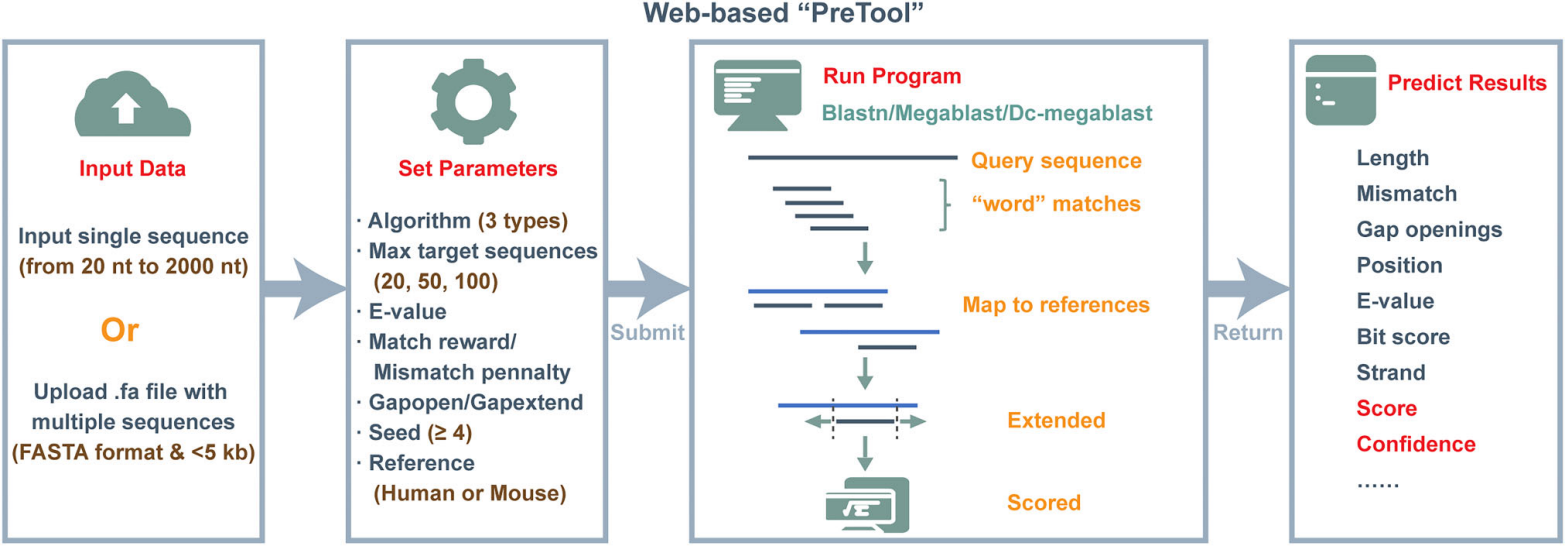
Workflow of the web-based prediction tool “PreTool”.

napRNA is a novel RNA type discovered only recently. Experimental methods and high-throughput sequencing techniques for identifying napRNA are time-consuming, labor-intensive, and costly. To achieve cost-effective napRNA identification, we developed a web-based prediction system named “PreTool” based on sequence similarity, designed to predict the potential of any target sequence to generate napRNA (Fig. 3). We firstly constructed reference libraries using experimentally validated napRNA sequences from humans and mice, respectively. Subsequently, we integrated three computational models from the BLAST algorithm—megablast, dc-megablast, and blastn—to perform sequence similarity analysis. “PreTool” allows users to flexibly select models and adjust parameters according to species type and sequence characteristics. Among these, megablast is optimal for sequence alignments between closely related species, particularly intra-species comparisons, with extremely high speed; dc-megablast is specifically designed for cross-species comparisons; blastn can be used for sequences with lower similarity. Default seed values differ across models, but all enforce a minimum threshold of 4. “PreTool” retains BLAST’s heuristic algorithmic workflow by identifying word-matches between query sequences and reference library, followed by extending alignments from these matched regions. In brief, users can balance alignment speed and accuracy by adjusting the seed value. Larger seed values enhance alignment speed and precision but may miss some distantly homologous sequences, whereas smaller seed values slow the alignment process but enable detection of more distantly related homologs. Furthermore, accurate queries can be achieved by flexibly adjusting match/mismatch scores and gap costs based on sequence conservation, while the output can be customized by specifying the E-value and the maximum number of target sequences. “PreTool” enables high-confidence predictions through scalable single-sequence and batch analysis capabilities. Users are allowed to paste a single sequence into the text box or click the button to upload a FASTA file containing multiple sequences, which will be aligned against human or mouse reference libraries, ultimately obtaining closely related napRNAs with detailed annotations.

### Exploring the associations between napRNAs and multiple interaction factors

**Figure 4.**
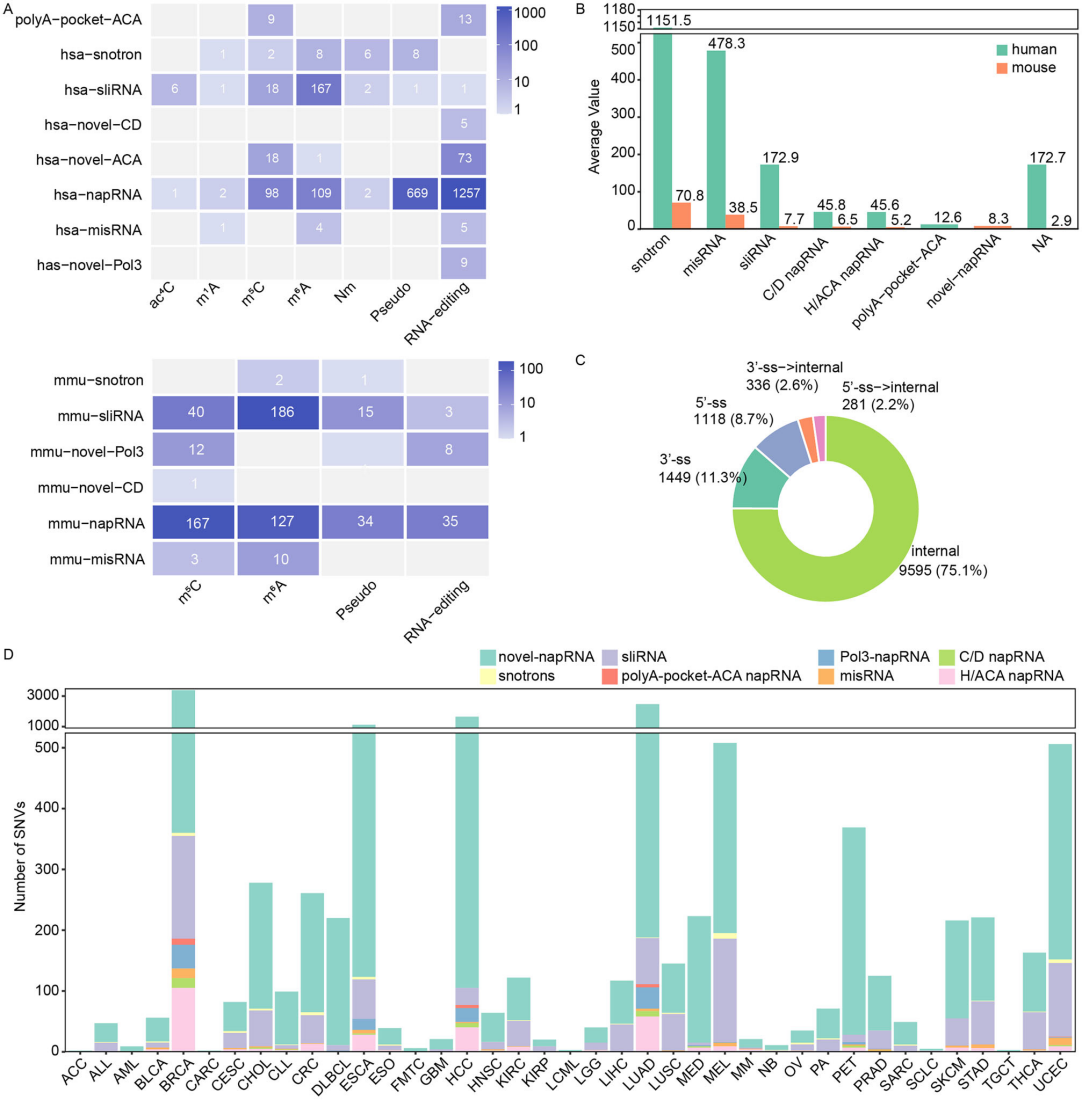
The associations between napRNAs and multiple interaction factors. (**A**) Statistics on the number of modification sites for various categories of napRNAs in human and mouse. (**B**) Statistics on RBP-binding events for various categories of napRNAs in human and mouse. (**C**) Distribution of SNVs within napRNAs and their 20 nt flanking regions. (**D**) Statistics on SNVs for different categories of napRNAs across various cancer types.

To further investigate the potential processing mechanisms and regulatory functions of napRNAs, we systematically performed association analyses between napRNAs and multiple interacting factors. Given the crucial roles of RNA modifications and RBPs in RNA splicing and functions [32–35], we collected 755 182 RNA modification sites and 106 614 858 RBP binding sites from RMBase [26] and starBase [27], comprising 453 650 human and 301 532 mouse modification sites, alongside 99 817 348 human and 6797 510 mouse RBP-binding sites. Subsequently, we defined ±20 nt flanking regions relative to napRNA boundaries and performed intersection analyses with both RNA modification sites and RBP binding events. This yielded 2497 human and 645 mouse high-confidence napRNA-RNA modification regulatory pairs, as well as 215 699 human and 53 294 mouse napRNA-RBP interactions respectively. We performed in-depth enrichment pattern analysis by specific RNA modification type and found that sliRNAs, snotrons, and misRNAs in both human and mouse are more likely to interact with m^6^A modification, whereas other types of napRNAs in human show stronger associations with RNA editing (Fig. 4A). Furthermore, enrichment analysis of RBP-binding events revealed that snotrons, misRNAs, and sliRNAs in both species exhibited stronger interactions with RBPs. Among these, snotron RNAs had the highest average number of interactions, with 1151.49 and 70.82 binding events per snotron RNA in human and mouse, respectively (Fig. 4B). These findings suggest that snotrons, misRNAs, and sliRNAs may play more diverse functional roles through m^6^A modification and specific RBPs.

Certain napRNAs have been found to be closely associated with a variety of human diseases, such as Prader–Willi syndrome [12, 13]. To investigate the clinical relevance between napRNAs and SNVs, we characterized the SNV mutation profiles of 12 779 napRNAs across eight distinct categories, including substitutions (Subs), deletions (Del), insertions (Ins), deletions–insertions (DelIns), and duplication (Dup). By investigating SNV clusters within napRNAs and their 20 nt flanking regions, we found that SNVs located within the 20 nt regions outside the ends of napRNAs were more abundant than those inside the napRNA boundaries (Fig. 4C). This may suggest that SNVs outside the processing boundaries of napRNAs play more important roles in napRNA processing and function. Furthermore, we characterized and visualized SNVs on various types of napRNAs across 39 cancer types. The results revealed that novel napRNAs and sliRNAs harbored the highest number of SNV sites across cancers, while Breast Invasive Carcinoma (BRCA) exhibited the highest number of napRNA-associated SNV events, with a total of 3385 cases (Fig. 4D). These findings may guide researchers in selecting more suitable napRNA research models and directions.

NapRNAdb provides a “Relationship” module to visualize the interplay between napRNAs and RNA modifications, RBP-binding events and SNVs. Users can retrieve interacting pair records through species and napRNA type specification queries.

### Case studies

#### Case 1: a concise case study of “Disease” module

**Figure 5.**
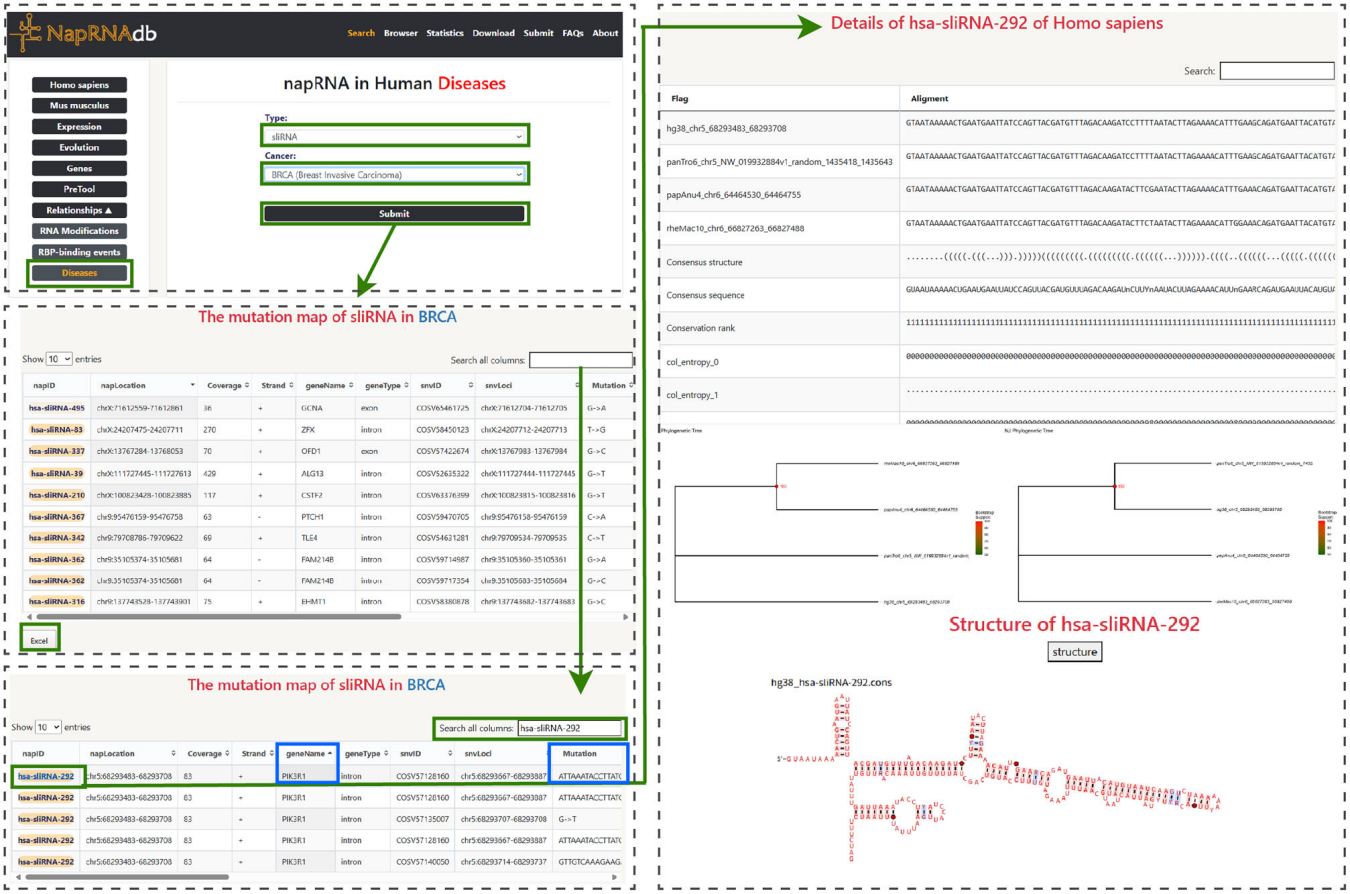
Demonstration of the “Disease” module using hsa-sliRNA-292 and BRCA (breast invasive carcinoma).

As shown in Fig. 5, querying the “Disease” module for “sliRNA” and “BRCA” returns 169 SNV records. The full dataset can be downloaded via the “Excel” button in the lower-left corner. Subsequent analysis shows that hsa-sliRNA-292 ranks among the top entries by SNV count. Searching “hsa-sliRNA-292″ in the upper-right search bar returns a detailed list of its five associated SNVs. According to the “geneName” and “geneType” columns, this napRNA originates from an intronic region of PIK3R1. The “Mutation” column specifies the nucleotide variations. Clicking the color-coded “napID” block leads to a detail page showing MSA, cross-species conservation, and secondary structure. Using prior knowledge of PIK3R1 and evolutionary conservation, potential functions of hsa-sliRNA-292 can be inferred. Secondary structure annotation also helps determine nucleotide exposure, supporting target identification and interaction analysis for functional exploration.

#### Case 2: a concrete example of napRNA interactome

**Figure 6.**
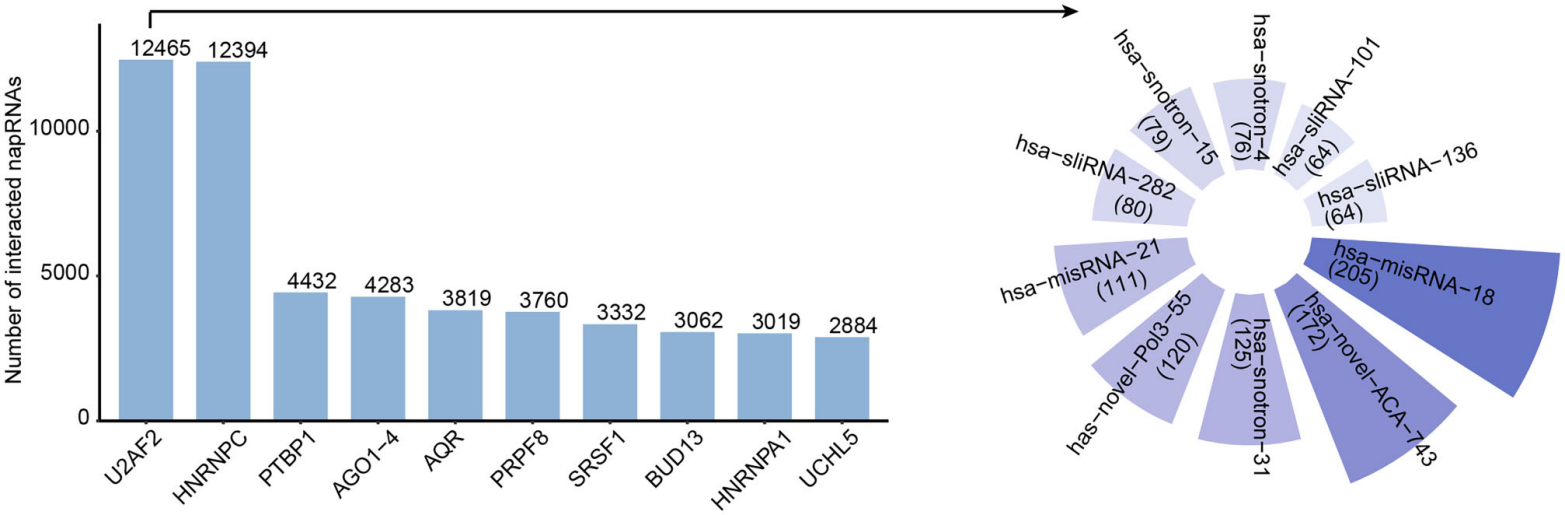
Interaction landscape between napRNAs and RBPs. The frequency-based ranking of top 10 RBPs and top 10 napRNA targets of the primary RBP.

We performed an in-depth analysis of the binding events between human RBPs and napRNAs and found that U2AF2 showed the highest binding frequency (Fig. 6), suggesting extensive and specific interactions. Previous studies have reported that U2AF2, as a core component of the spliceosome, can regulate alternative splicing, mRNA stability, and gene expression by interacting with ncRNAs such as circular RNAs (circRNAs) or lncRNAs, thereby contributing to disease pathogenesis [36, 37]. Notably, we identified a total of 12 465 binding events between U2AF2 and napRNAs. Among these, the most frequently bound napRNA was hsa-misRNA-18, with 205 binding events (Fig. 6). According to napRNAdb records, hsa-misRNA-18 originates from MIR221 gene, a miRNA host gene that is often overexpressed in cancers and acts as an oncogene. For instance, in liver cancer, MIR221 enhances tumor cell proliferation, migration, and invasion by binding to the 3′-UTR of target mRNAs, such as p27 and PTEN, inducing their degradation or translational inhibition [38]. These insights may contribute to uncovering the functional mechanisms by which the interaction between hsa-misRNA-18 and U2AF2 regulates tumorigenesis.

#### Case 3: example application of the “PreTool” Module

**Figure 7.**
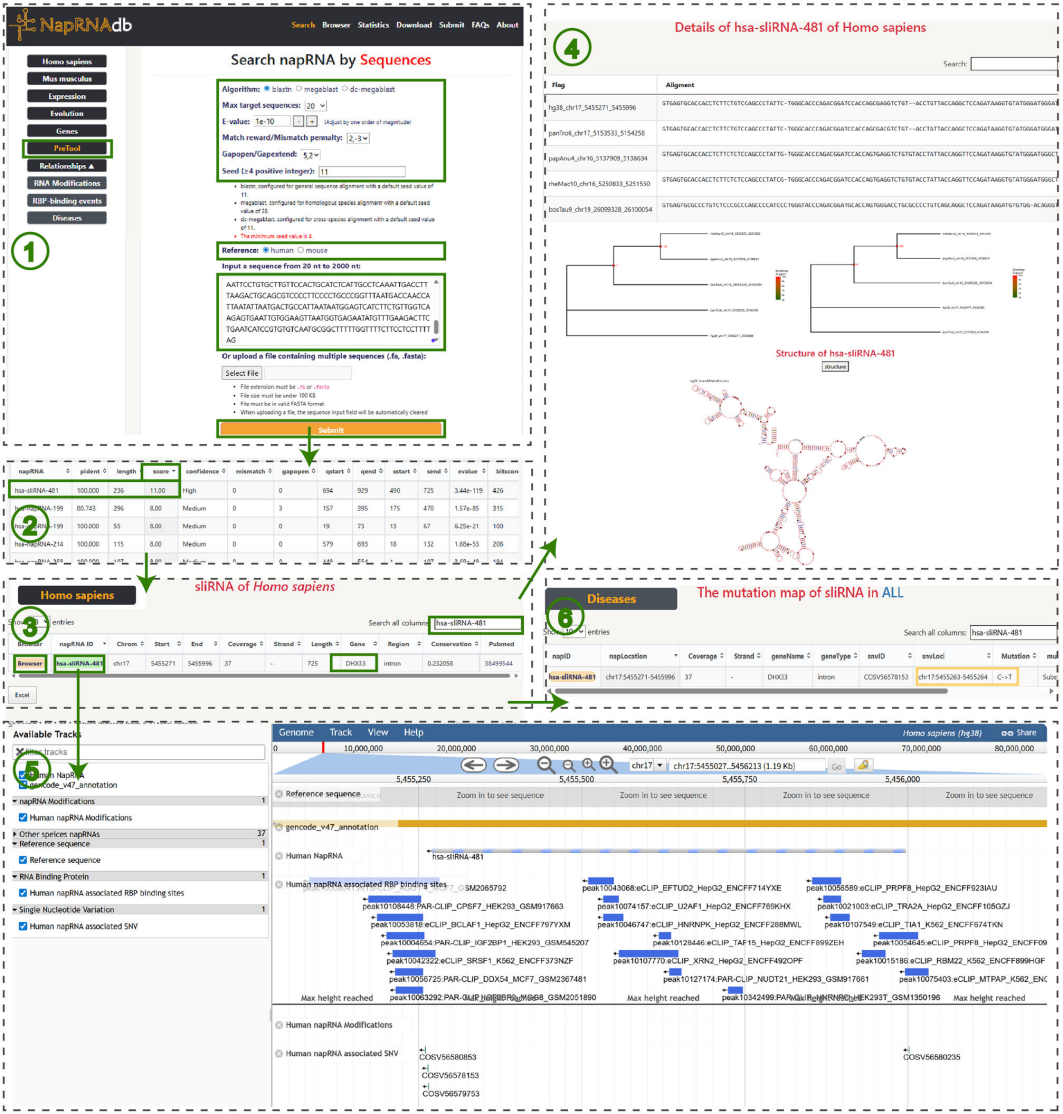
Demonstration of the “PreTool” module.

If researchers possess a specific sequence (Supplementary Materials) and wish to explore its potential association with napRNAs using NapRNAdb, they may input the sequence into the module’s text box or upload a file in “.fa/.fasta” format (sequence length limit: 20–2000 nt). Based on the species origin of the sequence, users can select either human or mouse reference databases via the “Reference” option and customize multiple parameters, including “Algorithm”, “Max_target_seqs”, “e_value”, “Match”, “Gap”, and “Seed”, to optimize search accuracy and efficiency. After configuring the settings, click the “Submit” button to initiate the analysis task (Fig. 7).

Upon completion, sort the result table in descending order based on the “score” column. The top-ranking entry, hsa-sliRNA-481, indicates the napRNA with the highest match to the query sequence. The “Homo sapiens” module enables retrieval of additional genomic information such as its genomic coordinates (chr17: 5455 271–5455 996) and host gene (DHX33). Furthermore, the system provides visualization of the phylogenetic tree and secondary structure of hsa-sliRNA-481. The integrated genome browser also displays hsa-sliRNA-481 together with its track signals including RBP-binding events and SNV sites, offering valuable clues for functional investigations. For instance, conserved loops and bulges may serve as binding sites for proteins or other RNAs, while predicted RBP-binding events can help infer functions related to stability or expression regulation [39]. Users may also explore associations between hsa-sliRNA-481 and specific diseases (e.g. acute lymphoblastic leukemia) through the “Disease” module, combining disease-associated mutation sites to further elucidate the potential role of hsa-sliRNA-481 in disease progression.

### Visualization of napRNAs and their interactome using the NapRNAdb genome browser

**Figure 8.**
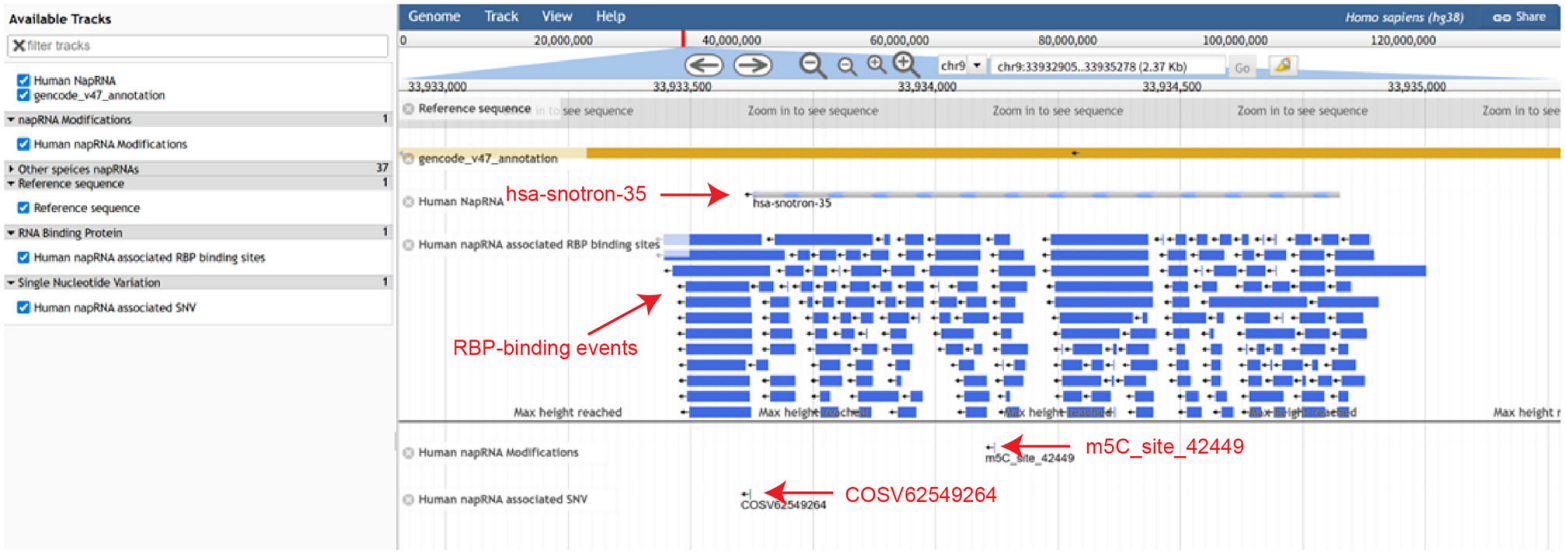
NapRNAdb genome browser visualize the genomic context of hsa-snotron-35 and its interactome.

To facilitate the visualization and in-depth exploration of various napRNAs, we provided the NapRNAdb genome browser based on JBrowse [30] (Fig. 8). This browser offers information on napRNA genomic coordinate and annotations, cross-species evolutionary conservation, as well as RNA modification sites, RBP-binding sites, and SNV sites interacting with napRNAs. In the query page of the browser, users can enter one genomic region of interest in the “Search Term” to obtain an integrated view of various genomic features. As shown in Fig. 8, the NapRNAdb browser was used to visualize the genomic context of hsa-snotron-35, revealing associated RNA modifications (m5C_site_42 449), various RBP-binding events (e.g. IGF2BPs, FTO), and SNV signals (COSV62549264).

### A web interface for submitting novel napRNA datasets

Despite current scarcity of napRNA research data, these molecules may play crucial roles in transcriptomic regulation. To continuously update and curate napRNA datasets, we developed a user-friendly web interface, named “Submit”, enabling researchers to submit arbitrary napRNA datasets online. This platform provides an accessible and comprehensive resource for the napRNA research community.

## Discussion and conclusions

Unlike sRNA-seq and conventional RNA-seq employed for identification and characterization of sRNAs [7–9], NAP-seq technology permits transcriptome-wide profiling of full-length napRNA sequences that are longer and structured, featuring diverse terminal modifications at single-nucleotide resolution [15]. This capability enables the revelation of distinct classes of structured ncRNAs exhibiting stable configurations [15]. Given the emerging nature of these long napRNAs, dedicated databases for managing these specialized datasets remain unavailable. Although Liu *et al.* introduced a supplementary webpage showcasing napRNAs identified in their NAP-seq methodology publication [15], its limited functionality proves inadequate for supporting researchers' requirements for systematic characterization and in-depth investigation of this novel RNA class.

Encouragingly, NapRNAdb, as the first international database dedicated specifically to NAP-seq-derived napRNAs, effectively addresses researchers' curiosity in systematically exploring napRNAs. By integrating napRNAs from published literature with those obtained through evolutionary conservation analysis, the database reveals transcriptome-wide landscapes of 8 distinct napRNA types across 40 species and probes their potential roles in gene regulation and diseases. Compared to other databases focusing on short napRNAs identified by conventional RNA-seq (such as miRbase [40] and Pol3Base [31]), the highlights of NapRNAdb include: (i) In a groundbreaking effort, it captures a class of NAP-seq-derived napRNAs that are longer, structured, and feature diverse terminal modifications, which are missed by conventional sequencing technologies; (ii) Unlike miRbase [40], it provides sequences and annotations of NAP-seq-derived napRNAs in human and mouse, coupled with provision of their expression profiles across diverse cell types and physiological states, delivering multidimensional data resources for interrogating napRNA functional mechanisms; (iii) Going beyond the original NAP-seq technology publication [15], it performs sequence conservation and homology analysis to extend the database’s coverage to napRNA transcriptomes across 38 additional species, and annotates a confidence level for each napRNA, substantially circumventing constraints imposed by elevated costs and technical demands of NAP-seq for large-scale investigations; (iv) In contrast to Pol3Base [31], it features an innovative and practical analysis module named “PreTool”, which enables interactive evaluation of the potential of any target sequence (not stored in the NapRNAdb) to be processed into napRNA and supports user-defined parameters, thereby establishing convenient experimental frameworks for investigating processing mechanisms and potential functions; (v) It also supplies information on cross-species conservation and secondary structures for these novel napRNAs, facilitating research into their biogenesis and functional roles; (vi) It features an integrated JBrowse genome browser, allowing users to directly inspect napRNAs along with their annotation tracks. (vii) Its in-depth analysis of the napRNA interactome offers critical insights into the roles of napRNAs in gene regulation and disease.

Collectively, NapRNAdb performs systematic integration and analysis of existing NAP-seq datasets to delineate napRNA landscapes across 40 species at the transcriptome level. The platform further establishes a web-based tool and multiple interactive interfaces, enabling comprehensive investigation of napRNAs through multidimensional examination of their landscapes, structural architectures, expression profiles, and functional potential. As the first integrated platform dedicated to napRNA research, NapRNAdb delivers novel conceptual frameworks and creates unprecedented investigative avenues for the comprehensive dissection of napRNAs and their potential functions.

## Future directions

With the rapid advancement of experimental methodologies and high-throughput sequencing technologies, transcriptome-wide discovery of novel RNAs has significantly accelerated. We aim to systematically enrich the database by integrating newly generated data. NapRNAdb will expand to include diverse napRNA types, detailed annotations, and broader taxonomic coverage. Through consistent curation and development of efficient bioinformatic workflows, we will enhance precise transcriptome-wide napRNA prediction. Our goal is to establish NapRNAdb as the premier and most comprehensive international resource for napRNA research.

## Supplementary Material

gkaf1100_Supplemental_File

## Data Availability

NapRNAdb is freely available at https://bioinformaticsscience.cn/naprnadb. All data files can be downloaded and used in accordance with the GNU Public License and the licenses of the primary data sources.
