# Supplementary material for: NapRNAdb: a multispecies repository and analytical platform for napRNA discovery and functional annotation
Source: Nucleic Acids Res. 2025 Nov 3;54(D1):D226–38. doi: 10.1093/nar/gkaf1100 (PMC12807620; doi:10.1093/nar/gkaf1100)
Supplement: gkaf1100_Supplemental_File [file gkaf1100_supplemental_file.pdf]

Supplementary Material

Title:

NapRNAdb: a multi-species repository and analytical platform for napRNA discovery and functional annotation

Authors:

Jiajia Xuan[1,*], Chunhua Xiao[1], Yonglei Luo[1], Shidong Tang[1], Junjie Pang[2], Zhirong Chen[3], Wanting Liu[1,*], Qing-Yu He[1,*]

Supplementary Materials

A queried sequence for case 3:
GTGAGTGCACCACCTCTTTAGCCTTGAACTTATCCCTTAAAAGCATGAGGTCTGA
GGATAATAAGTGGGGCTAGTACCTGTTACCAGGCTCCAGATAAGGTGTATGGGAT
GGGATACCCAGGTGCCAGATCAGCGCACTGAGGAGGTGCAGGAATAAAGTTTGT
ATTACTGCTTTTCAGATTATTTATTCACTCCAAAGCTTCTTAAAATACCTATGCAC
TTTTCTGACCCACACATATATCTATTTTTATATGGCTTGGCCATTCCAAAGTTGAG
GGATAGGGATGGATGCGACTTATTAGATAAATCTTTGTGGAAAATTTAGTGGAAG
AGCTATGTTGAATTGAGTAGGGTCATTAAATACTGGAATGATTGTCCAGACAGTG
ATCCTAAAGGGATGAGGGAGAGTATAAACCAAAGCTGCAAGGTAAACGAGTGAC
TTCTAAGATTATATACTGTACATAAAATATCAAAGTACCCAAAGTATGTATTATA
TACTGTACATAAAATATGAAAGTACCCAAACATTTATAATAAACTGTACATAAAA
TATCCCCAAGAAGGGAAGTAACAATTGCGAGGAATTTGGTATAATTATGGTGGGT
GATTATTTTTATACTGTATGTGCCAAAGCTTTACTACTGTGGAAAGACAGCTGTT
TTAATAAAAGATTTACATTCCACAAAAGAAAAATTCCTGTGCTTGTTCCACTGCA
TCTCATTGCCTCAAATTGACCTTTAAGACTGCAGCGTCCCCTTCCCCTGCCCGGTT
TAATGACCAACCATTAATATTAATGACTGCCATTAATAATGGAGTCATCTTCTGTT
GGTCAAGAGTGAATTGTGGAAGTTAATGGTGAGAATATGTTTGAAGACTTCTGAA
TCATCCGTGTGTCAATGCGGCTTTTTGGTTTTCTTCCTCCTTTTAG

Supplementary Tables

Table S1. The scoring rules of the comprehensive scoring system

Score	Identity	e-value	coverage
1	0%~40%	>1e-50	0%~40%
2	40%~60%	<=1e-50	40%~60%
3	60%~80%	<=1e-80	60%~80%
4	80%~100%	<=1e-110	80%~100%

Table S2. The scoring system for mammalian species

Score	Identity	E-value	Coverage
1	0%~70%	>1e-55	0%~70%
2	70%~80%	<=1e-55	70%~80%
3	80%~90%	<=1e-90	80%~90%
4	90%~100%	<=1e-125	90%~100%

Table S3. The scoring system for non-mammalian species

Score	Identity	E-value	Coverage
1	0%~70%	>1e-30	0%~40%
2	70%~80%	<=1e-30	40%~60%
3	80%~90%	<=1e-50	60%~80%
4	90%~100%	<=1e-70	80%~100%
